# Antimicrobial Susceptibility of Environmental Non-O1/Non-O139 *Vibrio cholerae* Isolates

**DOI:** 10.3389/fmicb.2018.01726

**Published:** 2018-08-02

**Authors:** Sivan Laviad-Shitrit, Yehonatan Sharaby, Ido Izhaki, Avi Peretz, Malka Halpern

**Affiliations:** ^1^Department of Evolutionary and Environmental Biology, The Faculty of Natural Sciences, University of Haifa, Haifa, Israel; ^2^Faculty of Medicine, Bar-Ilan University, Ramat Gan, Israel; ^3^Clinical Microbiology Laboratory, Baruch Padeh Medical Center, Tiberias, Israel; ^4^Department of Biology and Environment, The Faculty of Natural Sciences, University of Haifa, Haifa, Israel

**Keywords:** *V. cholerae*, antimicrobial susceptibility, resistance, antibiotics, chironomid, fish, waterbird, waterfowl

## Abstract

*Vibrio cholerae* serogroups O1 and O139 are the causative agents of cholera disease. There are more than 200 serogroups in this species that are termed *V. cholerae* non-O1/non-O139. Non-O1/non-O139 strains can cause gastroenteritis and cholera like diarrhea, wound infections, external otitis, and bacteraemia that may lead to mortality. Previous antimicrobial susceptibility studies were conducted mainly on O1/O139 serogroups and on clinical isolates. Our aim was to study and compare the antimicrobial susceptibilities of non-O1/non-O139 environmental strains isolated from chironomids, fish, and waterfowl. Significant differences were found in the antimicrobial susceptibilities between the environmental strains that were isolated from three different reservoir habitats. Significant increase in minimum inhibitory concentrations (MICs) of ampicillin and chloramphenicol was found in chironomid isolates from 2009 compared to those from 2005. *V. cholerae* isolates from different waterfowl species displayed the highest MIC values to chloramphenicol and trimethoprim-sulfamethoxazole (SXT), while chironomid isolates demonstrated the highest MIC values toward ampicillin. Isolates from fish and waterfowl showed high MIC values toward doxycycline. No significant differences were found between the MICs of isolates from the different waterfowl species. The percentage of antimicrobial resistance among *V. cholerae* isolates from waterfowl was the highest compared to the abundance of antimicrobial resistant isolates from chironomids or fish. The antimicrobial resistance genes can be carried on mobile genetic elements, thus, waterfowl may act as reservoirs for these elements and may spread them all over the globe. Data regarding treatment with antimicrobial agents toward *V. cholerae* non-O1/non-O139 serogroups is lacking and therefore further studies are needed.

## Introduction

*Vibrio cholerae* is a Gram-negative rod with more than 200 different serogroups. Only serogroups O1 and O139 are considered as the causative agents of the epidemic diarrheal disease cholera. Cholera is an endemic disease in many Asian and African countries. Non-O1/non-O139 strains share the same environmental niche with the pathogenic strains. They can cause intestinal infections such as gastroenteritis and cholera-like diarrhea, and also extra-intestinal infections such as wound infections, otitis media, external otitis, and bacteraemia that sometimes can cause mortality in humans (Sack et al., 2004; Bier et al., 2015; Kechker et al., 2017).

Antibiotics are not required to treat diarrheal cholera or diarrheal cholera-like symptoms. The main treatment for cholera is oral rehydration therapy. However, in severe cholera cases, a combination of oral rehydration therapy with antibiotic treatment is recommended since the antibiotics decrease the volume of the diarrhea (Kitaoka et al., 2011). Nevertheless, antimicrobial agent therapy is required for treating extra-intestinal infections caused by non-O1/non-O139 strains. For example, non-O1/non-O139 *V. cholerae* bacteremia is a life-threatening infection (Petsaris et al., 2010). Mortality occurred in 33% out of 350 non-O1/non-O139 bacteraemia cases (Deshayes et al., 2015). It was described that non-O1/non-O139 strains are usually susceptible to most antimicrobial agents (Petsaris et al., 2010; Ceccarelli et al., 2015). However, resistances of non-O1/non-O139 strains to aminoglycosides, ampicillin and carbapenems was reported (Ottaviani et al., 2003; Bier et al., 2015).

According to the guidelines of the Clinical and Laboratory Standards Institute (CLSI, 2018) there are some antibiotics that were recommended for treating *V. cholerae* infections (these recommendations do not distinguish between serogroups). The recommended antimicrobial agents are: ampicillin that acts as a cell growth inhibitor; azithromycin, doxycycline, and chloramphenicol that inhibit protein synthesis; and trimethoprim-sulfamethoxazole (SXT) that inhibits folic acid metabolism (CLSI, 2018).

Chironomids (*Diptera*, *Chironomidae*), also known as non-biting midges, are the most abundant insects in freshwater aquatic ecosystems (Laviad and Halpern, 2016). Chironomids were found as natural reservoirs of *V. cholerae* (Broza and Halpern, 2001; Halpern et al., 2004; Senderovich and Halpern, 2013).

Fish are also reservoirs of *V. cholerae*. *V. cholerae* was isolated from about 30 fish species (reviewed in Halpern and Izhaki, 2017). It was isolated mainly from healthy fish intestines; however, it was also detected in the fish gills, skin, kidney, liver, and brain tissues (Halpern and Izhaki, 2017). *V. cholerae* O1 and O139 were isolated from fish mucus and scales in Mozambique, from tilapia gills in Tanzania, and from two marine fish in India (du Preez et al., 2010; Kumar and Lalitha, 2013; Hounmanou, 2015). Moreover, many studies found correlations between the occurrence of cholera and fish consumption or handling (e.g., Acosta et al., 2001; Schürmann et al., 2002).

Although *V. cholerae* has been studied since 1884 (Koch, 1884), the mechanism that enables the bacteria to disseminate around the globe is still an enigma. Halpern et al. (2008) suggested that waterfowl can be the vector responsible for this dissemination. Additionally, they explained that waterfowl can either acquire the bacterium from zooplankton (chironomids or copepods) or from fish consumption. Fish can get infected with *V. cholerae* via zooplankton consumption (chironomids or copepods) (Halpern et al., 2008). Recently, we have demonstrated that *V. cholerae* can be identified from wild waterfowl intestines (Laviad-Shitrit et al., 2018) and that great cormorants got infected with *V. cholerae* after they were fed with tilapia fish that were naturally infected with *V. cholerae* (Laviad-Shitrit et al., 2017).

As far as we know, data regarding susceptibility of different *V. cholerae* non-O1/non-O139 environmental serogroups is very rare. Knowledge of their antimicrobial resistance is of global health interest as they are dispersed all over the world and share the same niche with the pathogenic strains (Halpern and Izhaki, 2017; Laviad-Shitrit et al., 2017, 2018).

Here we studied and compared the minimum inhibitory concentration (MIC) of different environmental *V. cholerae* non-O1/non-O139 serogroups that were isolated from three different reservoirs of *V. cholerae*: chironomid egg masses and fish and waterfowl intestines.

## Materials and Methods

### *Vibrio cholerae* Isolates

Isolates were obtained from previous studies. 40 isolates were obtained from chironomid egg masses (Senderovich et al., 2008; Shaked, 2011), 49 isolates from fish intestine samples (Senderovich et al., 2010; Laviad-Shitrit et al., 2017), and 47 isolates from waterfowl intestine samples (Laviad-Shitrit et al., 2017, 2018). Details regarding the sampling sites, sampling dates and the identity of the sampled fish and waterfowl species, can be found in Supplementary Table S1.

The isolation and Identification of *V. cholerae* was described in each of the references that were mentioned above. Briefly, *V. cholerae* was isolated directly on Thiosulfate-citrate-bile salts-sucrose (TCBS) agar plates (Difco, United States), without any enrichment (e.g., Laviad-Shitrit et al., 2017). Isolates’ identities were verified by multiplex PCR assay that amplified the *ompW* gene that encodes an outer membrane protein specific to *V. cholerae* and *ctxA* gene that encodes the cholera enterotoxin subunit A, according to Nandi et al. (2000). The identity of O1 and O139 serogroups was examined by slide agglutination with the use of two specific antisera: (1) a poly antiserum specific for O1 surface antigen (Difco), and (2) an antiserum specific for O139 surface antigen (Ministry of Health, Israel). Moreover, *V. cholerae* isolates from waterfowl underwent somatic O antigen serogrouping (Laviad-Shitrit et al., 2018). The procedure of somatic O antigen serogrouping is described in details in Shimada et al. (1994). The non-O1/non-O139 serogroups identities are described in Supplementary Table S2.

### Determination of Minimum Inhibitory Concentration (MIC)

All isolates were tested for their antimicrobial susceptibilities using the *E*-test gradient. Each isolate was classified as resistant (R), intermediately resistant (I), or susceptible (S), according to the guidelines of the Clinical and Laboratory Standards Institute (Supplementary Table S3 and CLSI, 2018). Isolates were spread on CHROMagar plate (HyLaboratories, Rehovot, Israel) and then incubated at 37°C for 24 h. Colonies were suspended in 0.45% NaCl solution to 0.5 McFarland turbidity and subjected to MIC test strip (Liofilchem S.r.l., Italy) on Mueller Hinton agar plates (BD Diagnostics, Sparks, MD, United States). The antibiotics that were tested were chosen according to the guidelines of the Clinical and Laboratory Standards Institute (CLSI, 2018): ampicillin, azithromycin, chloramphenicol, doxycycline, and trimethoprim-sulfamethoxazole (hereinafter SXT). Plates were incubated for 24 h at 37°C. MIC was calculated for each antibiotic as the lowest concentration of the antibiotic at which the zone of inhibition intersected the strip.

### Statistical Analysis

Repeated measures ANOVA with Bonferroni *post hoc* test was used to determine the differences between the MICs of the five antimicrobial agents (*n* = 147 strains). Data sphericity was not assumed (Mauchly’s test: *p* < 0.001) and Huynh-Feldt correction was applied (*E* = 0.375). One-way ANOVA tests with Tukey HSD *post hoc* were used to determine the differences between MICs of *V. cholerae* strains isolated from chironomids, fish, and waterfowl, and from different waterfowl species (herons, egrets, and cormorants). Independent samples Student *t*-tests were applied to compare the MICs for *V. cholerae* strains that were isolated from chironomids at different years (2005 and 2009).

## Results

**FIGURE 1:**
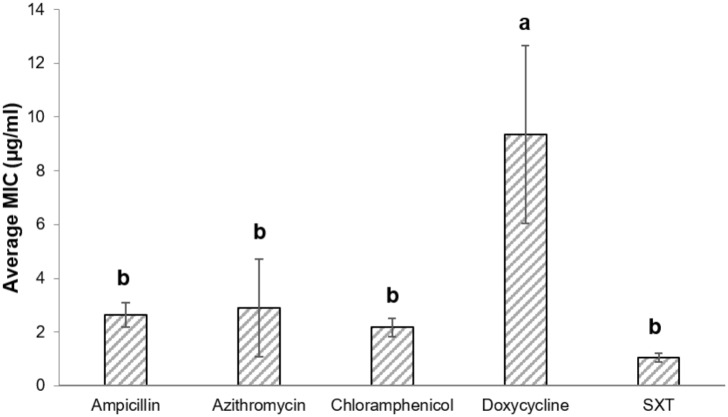
MIC (average ± std. error) of the studied antimicrobial agents toward all *V. cholerae* strains. Ampicillin (*n* = 146), azithromycin (*n* = 141), chloramphenicol (*n* = 146), doxycycline (*n* = 144); SXT (*n* = 146). Bars with different letters are significantly different by repeated measures ANOVA with Bonferroni *post hoc* test (*p* < 0.05). SXT, Trimethoprim (TMP) and sulfamethoxazole.

Antibiotic susceptibilities of 147 *V. cholerae* environmental non-O1/non-O139 strains were studied for five antimicrobial agents that are used to treat cases of *V. cholerae* infections. The strains were isolated from: (i) chironomid egg masses (50 strains; Senderovich et al., 2008; Shaked, 2011), (ii) fish intestines (50 strains; Senderovich et al., 2010; Laviad-Shitrit et al., 2017), and (iii) waterfowl intestines (47 strains; Laviad-Shitrit et al., 2018). Overall, significant differences were observed in *V. cholerae* sensitivities to the different antimicrobial agents [Repeated measures ANOVA: *F*_(1.5,205.8)_ = 4.1, *p* = 0.029]. MIC values of doxycycline were the highest, with an average MIC of 9.34 ± 3.31 μg/ml (**Figure 1**) and MIC_90_ of 8.0 μg/ml (**Table 1**). No significant differences were observed between the MIC values of the other four antimicrobial agents that were studied (*p* > 0.05).

**Table 1:** MIC_90_ and MIC_50_ values (μg/ml) for all *V. cholerae* isolates.

Source of isolation	Chironomids egg	Fish	Waterfowl	All strains
	masses (*n* = 50)	(*n* = 50)	(*n* = 48)	(*n* = 148)
**Antimicrobial Agent**				
Ampicillin	12.40 (1.25)	1.00 (0.25)	1.75 (0.38)	12.00 (0.44)
Azithromycin	1.10 (0.38)	2.00 (0.38)	3.00 (1.00)	2.00 (0.50)
Chloramphenicol	1.50 (1.00)	2.00 (0.75)	12.00 (1.50)	4.00 (1.00)
Doxycycline	1.00 (0.50)	16.00 (1.50)	14.40 (2.00)	8.00 (0.75)
SXT^∗^	0.75 (0.19)	1.50 (0.19)	5.00 (1.50)	3.00 (0.19)

*The values in parentheses are MIC_*50*_. ^∗^SXT, Trimethoprim (TMP) and sulfamethoxazole*.

**FIGURE 2:**
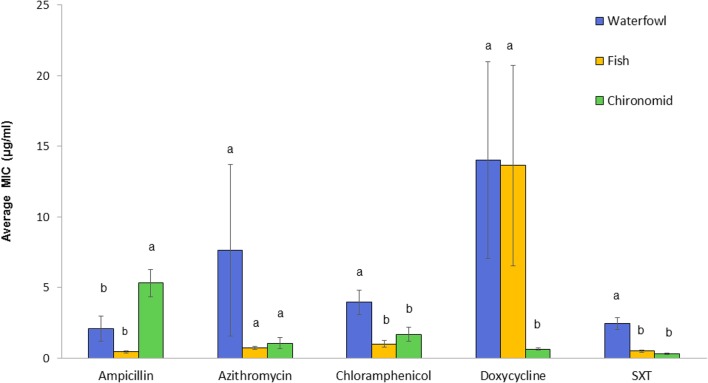
Comparative analysis of MICs (average ± std. error) for each of the studied antimicrobial agents toward *V. cholerae* strains that were isolated from the three environmental reservoirs (waterfowl *n* = 47, fish *n* = 50, and chironomids *n* = 50). Bars with different letters are significantly different by One-way ANOVA with Tukey HSD test (*p* < 0.05). SXT, Trimethoprim (TMP) and sulfamethoxazole.

*Vibrio cholerae* strains exhibited substantial variations in their susceptibilities toward the tested antimicrobial agents (**Figure 2**). High differences were found in the ampicillin MIC_90_ values between isolates from chironomid egg masses (12.4 μg/ml), from waterfowl species (1.75 μg/ml), and from fish (1 μg/ml). Isolates from fish and waterfowl intestine samples that were exposed to doxycycline showed high MIC_90_ values (16.0 and 14.4 μg/ml, respectively) compared to the isolates from chironomids (only 1.0 μg/ml). Additionally, isolates from waterfowls showed the highest MIC_90_ values after exposure to chloramphenicol and SXT (12.0 and 5.0 μg/ml, respectively) compared to isolates from fish (2.0 and 1.5 μg/ml, respectively) and from chironomid egg masses (1.5 and 0.75 μg/ml, respectively) (**Table 1**).

One-way ANOVA revealed significant differences in the average MIC of ampicillin, chloramphenicol, doxycycline, and SXT between *V. cholerae* strains that were isolated from chironomids, fish, and waterfowl; ampicillin [*F*_(2,__143)_ = 11.03, *p* < 0.001], chloramphenicol [*F*_(2,__143)_ = 7.11, *p* < 0.001], doxycycline [*F*_(2,__141)_ = 8.52, *p* < 0.001], and SXT [*F*_(2,__143)_ = 23.21, *p* < 0.001]. No significant difference was observed between MIC values of azithromycin (*p* = 0.24) (**Figure 2**). *V. cholerae* isolated from waterfowl were significantly more resistant to SXT and chloramphenicol with average MIC concentrations of 2.44 ± 0.42 and 3.95 ± 0.86 μg/ml, respectively (**Figure 2**). In contrast, *V. cholerae* isolates from chironomids were more resistant to ampicillin with average MIC value of 5.33 ± 0.98 μg/ml and a MIC_90_ value of 12.4 μg/ml (**Table 1**).

**FIGURE 3:**
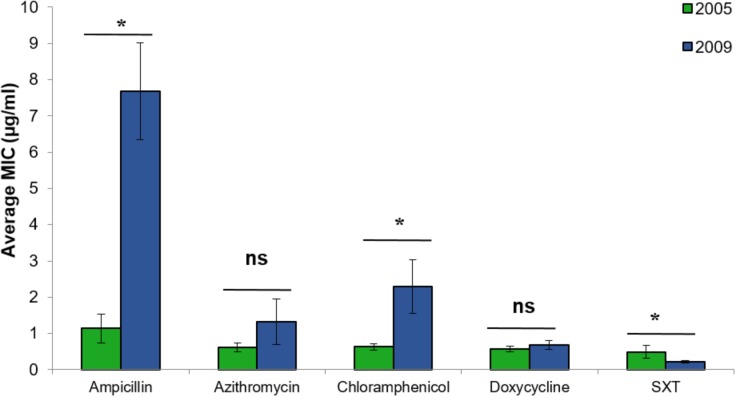
Comparative analysis of the MIC (average ± std. error) for each of the studied antimicrobial agents toward *V. cholerae* strains that were isolated from chironomids in 2005 (green bars, *n* = 18), and 2009 (blue bars, *n* = 32). Asterisks represent significant differences in MICs by Student’s *t*-test (*p* < 0.05). ns, no significant difference; SXT, Trimethoprim (TMP) and sulfamethoxazole.

*Vibrio cholerae* strains isolated from chironomids during 2009 showed significantly higher MICs of ampicillin, chloramphenicol, and SXT compared with the strains that were isolated during 2005 (**Figure 3**) [Students *t*-test: ampicillin: *t*_(36.3)_ = 4.67, *p* < 0.001; chloramphenicol: *t*_(31.9)_ = 2.25, *p* = 0.032; SXT: *t*_(48)_ = 2.05, *p* = 0.046]. No significant differences were found between *V. cholerae* strains isolated from chironomids at the Kishon River vs. WSP (independent samples *t*-tests: *p* > 0.05).

**FIGURE 4:**
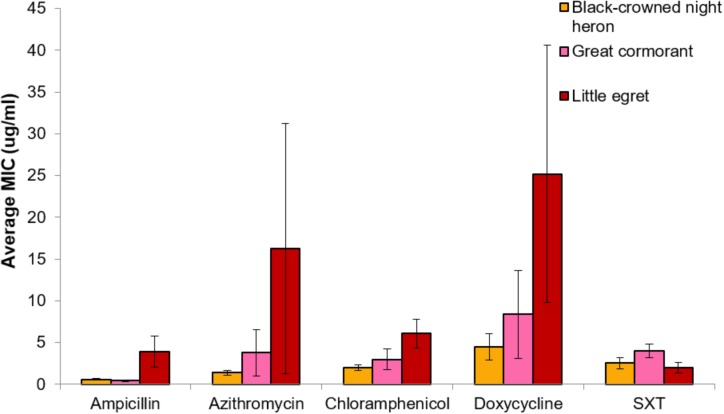
Comparative analysis of MIC (average ± std. error) for each of the studied antimicrobial agents toward *V. cholerae* strains that were isolated from the three waterfowl species [black-crowned night herons (orange, *n* = 21), great cormorants (pink, *n* = 5), and little egrets (red, *n* = 22)]. No significant differences were found between MIC concentrations of the different strains that were isolated from different waterfowl species (*p* > 0.05).

No significant differences in MIC concentrations were found between strains isolated from the different waterfowl species (herons, egrets, and cormorants, *p* > 0.05) (**Figure 4**). Four strains that were isolated from two different individual herons were resistant even to the highest concentration of azithromycin that was studied (256 μg/ml) (**Table 2**).

**Table 2:** Cumulative distribution of the MIC values for all the studied strains.

Cumulative values (%) of *V. cholerae* strains that were inhibited at the indicated concentrations (μg/ml)^∗∗^
**Concentration (μg/ml)**	**0.008**	**0.032**	**0.064**	**0.125**	**0.25**	**0.5**	**1**	**2**	**4**	**6**	**8**	**12**	**16**	**24**	**32**	**256**

Ampicillin	2.7	7.5	15.8	21.9	41.1	57.5	76.7	82.2	85.6	86.3	87.0	95.2	97.9	98.6	100.0	
Azithromycin		0.7	2.8	11.8	36.1	56.9	74.3	89.6	94.4	95.1	95.1	96.5	97.2	97.2	97.2	97.9
Chloramphenicol		0.7	6.2	8.9	18.5	34.9	67.8	81.5	91.1	93.2	93.8	95.2	98.6	100.0		
Doxycycline			2.8	4.9	14.6	35.4	56.3	70.8	84.0	86.1	90.3	92.4	95.8	96.5	97.9	100.0
^∗^SXT	3.4	4.8	23.3	32.2	56.8	66.4	76.7	84.9	96.6	97.9	98.6	100.0				

*MIC_*50*_ (marked in pale gray) and MIC_*90*_ (marked in dark gray) can be read directly from this table*.

*^∗^SXT, Trimethoprim (TMP) and sulfamethoxazole*.

*^∗∗^Not all concentrations that were studied are represented in this table*.

Significant differences were observed between the average MIC values of SXT of strains that belonged to different serogroups [*F*_(3,__38)_ = 4.00, *p* = 0.014]. Serogroup O6 showed the highest MIC of SXT, with a MIC_50_ of 4 μg/ml and MIC_90_ of 5.58 ± 1.74 μg/ml. No significant differences in MICs of the other antibiotics were observed between serogroups (Supplementary Table S2).

## Discussion

Antimicrobial agent effectiveness is decreasing due to a significant increase of antimicrobial resistance in bacteria. Medicine achievements in major surgery, organ transplantation, and treatment of preterm babies, cancer chemotherapy, and many other clinical procedures cannot be successful without effective antibiotic treatments (Laxminarayan et al., 2013). It was estimated that bacteria resistant to antimicrobial compounds kill 25,000 people annually in Europe. In the United States, the estimated numbers for sickness and death due to antibiotic-resistant bacterial strains are 2 × 10^6^ and 2.3 × 10^4^ people every year (CDC, 2013).

It was found that different serogroups of *V. cholerae* use multidrug efflux pumps in order to export chemically and structurally unrelated components such as antibiotics (Paulsen et al., 1996), e.g., tetracycline, Norfloxacin, ciprofloxacin, doxorubicin, chloramphenicol, and nalidixic acid (Kitaoka et al., 2011). Moreover, antibiotic resistance can occur in *V. cholerae* via spontaneous mutations, horizontal gene transfer, and by conjugative plasmids (Kitaoka et al., 2011). *V. cholerae* strains that are resistant to the recommended antimicrobial treatment compounds (e.g., co-trimoxazole, chloramphenicol, sulphonamides, and nalidixic acid), were reported in South Asia and Africa. Resistance to cotrimoxazole and furazolidone in sub Saharan Africa and Bangladesh is increasing while resistance to tetracycline varies from year to year (Laxminarayan et al., 2013).

Antimicrobial susceptibilities studies of *V. cholerae* are mainly conducted on O1 and O139 clinical strains. Studying environmental strains is important because humans are usually infected from the environment. Here we studied antimicrobial susceptibilities of environmental *V. cholerae* non-O1/non-O139 strains that were isolated from chironomids, fish, and waterfowl species that are considered their natural reservoirs and vectors. Pathogenic *V. cholerae* strains share the same niche with the non-pathogenic strains as was found for *V. cholerae* in fish and waterfowl (Halpern and Izhaki, 2017; Laviad-Shitrit et al., 2018). Thus, resistance to antimicrobial compounds can be transferred from the non-pathogenic to the pathogenic strains inside their mutual host.

Isolates from waste water were found to be significantly more resistant to antimicrobial compounds relative to surface water. Waste water facilities are considered as hotspots for horizontal resistance gene transfer (Bouki et al., 2013). *Acinetobacter* strains isolated from waste water were resistant to amoxicillin/clavulanic acid, chloramphenicol, and rifampin, but not to ciprofloxacin, colistin, gentamicin, rifampin, sulfisoxazole, and trimethoprim (Zhang et al., 2009). Amoxicillin, ciprofloxacin, tetracycline, and sulfamethoxazole residues were detected in raw and treated waste water (Novo et al., 2013). In the current study, chironomid egg masses were sampled from a waste water stabilization plant. Isolates from chironomids showed higher ampicillin MIC values compared to waterfowl and fish isolates. Nevertheless, only one isolate was resistant to ampicillin and two were resistant to azithromycin (**Table 3**). *V. cholerae* is embedded in a biofilm within the gelatinous matrix that surrounds the egg mass, and thus it is probably protected from exposure to the antimicrobial compounds that are present in the waste water. Moreover, significant differences were obtained in MIC values of ampicillin, chloramphenicol, and SXT between the 2 years of sampling (2005 and 2009).

**Table 3:** Percentage of isolates that were found susceptible (S), intermediate (I), or resistant (R) to different antimicrobial agents in accordance to the Clinical and Laboratory Standards (CLSI, 2018; see also Supplementary Table S3).

Source of isolation	Chironomids *n* = 50			Fish *n* = 50			Waterfowl *n* = 48		
	S	I	R	S	I	R	S	I	R
Ampicillin	67.3	30.6	2.0	100.0	0.0	0.0	93.3	4.5	2.2
Azithromycin	95.8	0.0	4.2	96.0	0.0	4.0	77.8	0.0	22.2
Chloramphenicol	93.9	6.1	0.0	98.0	2.0	0.0	88.9	11.1	0.0
Doxycycline	100.0	0.0	0.0	74.0	14	12.0	77.8	11.1	11.1
SXT^∗^	97.9	0.0	2.0	98.0	0.0	2.0	55.6	0.0	44.4

*^∗^SXT, Trimethoprim (TMP) and sulfamethoxazole*.

Since 1940, antimicrobial agents have been added to animals’ food in order to reduce production costs and improve growth (Aarestrup, 2000). To date there is still a massive use of antimicrobial agents in animals’ food. In Israel, fish food in fish ponds is usually supplemented with one of the following antimicrobial compounds: oxytetracycline hydrochloride, sulfadiazine trimethoprim, and florfenicol. When transferring juvenile fish (weighing up to 100 g) between pools, the water is supplemented with oxytetracycline hydrochloride that is an analog of tetracycline (doxycycline) and is also used in humans (An et al., 2015). Indeed, the MIC_90_ of doxycycline in the fish isolates was the highest compared to the isolates from the other environmental reservoirs tested in the current study (**Table 1**). Isolates that were resistant to doxycycline were isolated in the current study from the following fish pond species; tilapia, grass carps, and Jordan mouthbrooder (**Table 2** and Supplementary Table S1). Although there is a minor difference between the different antimicrobial agents used for humans or animals, in some countries the amounts of antimicrobial agents that are used in agriculture and aquaculture are four times higher than that used in medicine (Laxminarayan et al., 2013).

Different antimicrobial drug resistance mechanisms were found in *V. cholerae* strains (reviewed in Kitaoka et al., 2011). For example, antimicrobial resistance can be carried on mobile genetic elements like conjugative plasmids, SXT elements and integrons. Integrating conjugative elements (ICEs) are transferred by cell contact and then integrate into the chromosome. The first Integrating conjugative element carrying antibiotic resistance genes was described in O139 strain isolated in India, in 1992 (Waldor et al., 1996). This ICE was named SXT element as it carried genes for SXT resistance (Beaber et al., 2004; Kitaoka et al., 2011). Since then, many strains that acquired SXT elements were detected all around the globe (Hochhut et al., 2001; Burrus et al., 2006; Kitaoka et al., 2011). Interestingly, in the current study we found that the percentage of antimicrobial resistant *V. cholerae* isolates from waterfowl intestines was the highest, compared to antimicrobial resistant isolates from chironomids and fish (**Table 3**). Thus, it may be that waterfowl act as reservoirs and vectors of *V. cholerae* strains which carry resistant conjugative elements and that waterfowl are spreading these genetic elements, globally.

## Conclusion

Studying the susceptibility of environmental pathogenic bacteria to antimicrobial agents is very important because *V. cholerae* causes disease originating from human exposure to environmental strains. Waterfowl species may be the vectors by which antimicrobial resistant strains and or resistant conjugative elements are spread all over the globe. Further study is needed to understand the bacterial antimicrobial resistance profiles and the dissemination of antimicrobial resistance strains via waterfowl.

## Author Contributions

SL-S, II, AP, and MH conceived and designed the experiments. SL-S and AP performed the experiments. AP, II, and MH contributed reagents, materials, and analysis tools. SL-S and YS analyzed the data. SL-S and MH wrote the paper. All authors discussed the results, reviewed, and commented.

## Conflict of Interest Statement

The authors declare that the research was conducted in the absence of any commercial or financial relationships that could be construed as a potential conflict of interest.
