# Supplementary material for: Antimicrobial Susceptibility of Environmental Non-O1/Non-O139 Vibrio cholerae Isolates
Source: Front Microbiol. 2018 Aug 2;9:1726. doi: 10.3389/fmicb.2018.01726 (PMC6083052; doi:10.3389/fmicb.2018.01726)
Supplement: Supplementary file 1 [file Table_1.pdf]

Table S1. Detailed information regarding *V. cholerae* strains that were studied in the current work.

Habitat	Common name (species name)	No. of isolates	Location, sampling date	Reference
	**Fish**			
Fish pond	Jordan mouthbrooder (*Astatotilapia flaviijosephi*)	1	Nir David, northern Israel, 2007	(Senderovich et al., 2010)
	Tilapia spp.	3	Nir David, northern Israel, 2007	(Senderovich et al., 2010)
	Redbelly tilapia (*Coptodon zillii*)	2	Nir David, northern Israel, 2007	(Senderovich et al., 2010)
	Grass carp (*Ctenopharyngodon idella*)	3	Atlit, northern Israel, 2008	(Senderovich et al., 2010)
	Flathead grey mullet (*Mugil cephalus*)	1	Nahalal, North Israel, October 2008	(Senderovich et al., 2010)
	Mango tilapia (*Sarotherodon galilaeus*)	5	Kfar Rupin, eastern Israel, 2008	(Senderovich et al., 2010)
	Hybrid tilapia (*Oreochromis niloticus* X *Oreochromis aureus*)	31	Nir David, northern Israel, 2013-2014	(Laviad-Shitrit et al., 2017)
The Sea of Galilee	Jordan himri (*Carasobarbus canis*)	1	2008	(Senderovich et al., 2010)
	Jordan barbell (*Luciobarbus longiceps*)	1	2008	(Senderovich et al., 2010)
Mediterranean Sea	Pinecone soldierfish (*Myripristis murdjan*)	1	Akko, northern Israel, 2008	(Senderovich et al., 2010)
River	Blue tilapia (*Oreochromis aureus*)	1	Asi stream, Nir David, northern Israel, 2009	(Senderovich et al., 2010)
	**Waterfowl**			
Fish pond	Great cormorant (*Phalacrocorax carbo*)	5	Ma'agan Michael, Beit She'an valley, Israel, 2014	(Laviad-Shitrit et al., 2017)
	Black-crowned night heron (*Nycticorax nycticorax*)	21	Ma'agan Michael, Israel, 2014	(Laviad-Shitrit et al., 2018)
	Little egret (*Egretta garzetta*)	21	Ma'agan Michael, Israel, 2014	(Laviad-Shitrit et al., 2018)
	**Chironomid egg masses**			
River and Waste Stabilization Pond (WSP)	Chironomid (*Chironomus* sp.)	18	Tivon WSP and Kishon River, northern Israel, 2005	(Senderovich et al., 2008)
	Chironomid (*Chironomus* sp.)	32	Tivon WSP, northern Israel, 2009	(Shaked, 2011)
