# Supplementary material for: Antimicrobial Susceptibility of Environmental Non-O1/Non-O139 Vibrio cholerae Isolates
Source: Front Microbiol. 2018 Aug 2;9:1726. doi: 10.3389/fmicb.2018.01726 (PMC6083052; doi:10.3389/fmicb.2018.01726)
Supplement: Supplementary file 2 [file Table_2.pdf]

Table S2. *V. cholerae* serogroups identified from Great cormorant (C), little egret (E), and black-crowned night heron (N) intestine samples. The numbers after the letters C E or N, represent the rank number of each individual bird from which *V. cholerae* was successfully isolated (*V. cholerae* was not isolated from all the sampled birds). Numbers in parentheses indicate the number of isolates. Data from Laviad-Shitrit et al., (2017); Laviad-Shitrit et al., (2018).

**Great cormorant**	C2						
**Serogroup identity (number of isolates)**	O36 (n=5)						
**Little egret**	E4	E7	E9	E10	E11		
**Serogroup identity (number of isolates)**	O13 (1)<br>O16 (1)<br>O36 (1)<br>O128 (1)	O171 (9)	O40 (1)	O6 (2)<br>O21 (1)<br>O123 (1)<br>O193 (2)	O36 (1)		
**Black-crowned night heron**	N1	N2	N3	N4	N5	N6	N7
**Serogroup identity (number of isolates)**	O94 (1)	O39 (1)<br>O94 (3)	O65 (1)<br>O93 (1)<br>O123 (1)	O6 (3)<br>O9 (1)<br>O18 (1)<br>O33 (1)<br>O85 (1)	O8 (1)<br>O93 (2)<br>O103 (1)<br>O195 (1)	O125 (1)<br>O126 (2)	O39 (1)<br>O103 (1)
