# Supplementary material for: Antimicrobial Susceptibility of Environmental Non-O1/Non-O139 Vibrio cholerae Isolates
Source: Front Microbiol. 2018 Aug 2;9:1726. doi: 10.3389/fmicb.2018.01726 (PMC6083052; doi:10.3389/fmicb.2018.01726)
Supplement: Supplementary file 3 [file Table_3.pdf]

Table S3. The standard values for MIC interpretive criteria.

Antimicrobial class	Antimicrobial agent	MIC (µg/ml) Interpretive Criteria		
		**S**	**I**	**R**
**β-lactam rings**	Ampicillin	≤8	16	≥32
**Macrolides**	Azithromycin	≤2	-	-
**Tetracyclines**	Doxycycline	≤4	8	≥16
**Folate pathway inhibitors**	Trimethoprim (TMP) and Sulfamethoxazole (SXT)	≤2	-	≥4
**Phenicols**	Chloramphenicol	≤8	16	≥32

S – susceptible, I – intermediate, and R – resistant; data from (CLSI, 2018).
